# Evaluation of Male Gonadal Radiation Dose in Abdominal X-ray Examinations: The Impact of Radiographic Projections and Kilovoltage Peak (kVp)

**DOI:** 10.7759/cureus.76175

**Published:** 2024-12-22

**Authors:** Inayatullah Shah Sayed, Nurul Syazwina Yusaini

**Affiliations:** 1 Department of Diagnostic Imaging and Radiotherapy, Kulliyyah of Allied Health Sciences, International Islamic University Malaysia, Kuantan Campus, Kuantan, MYS

**Keywords:** abdominal radiography, absorbed dose, esd, male gonad dose, nanodot osld

## Abstract

In abdominal X-ray examinations, radiosensitive organs such as the gonads within or near the imaging region are at risk of radiation exposure. Minimizing the dose to these organs is crucial to reducing unnecessary radiation. This study utilized optically stimulated luminescence dosimeters (OSLDs) to measure the radiation dose to the male gonads at varying kilovoltage peak (kVp) settings while keeping the milliampere-seconds (mAs) constant across different radiographic projections.

A Siemens Multix Top X-ray system (Siemens Healthcare Inc., Erlangen, Germany) was used for imaging, with three kVp settings (81, 83, and 85) and a fixed exposure of 32 mAs. The study employed a RANDO phantom (The Phantom Laboratory, Salem, New York, USA) to simulate human anatomy. The entrance surface dose (ESD) was recorded by placing an OSLD on the phantom's surface corresponding to the gonad location. In contrast, the absorbed dose was measured by positioning the OSLD at the gonadal region inside the phantom. Six abdominal projections were evaluated: anteroposterior (AP) supine, posteroanterior (PA) prone, lateral, AP erect, lateral decubitus, and dorsal decubitus. All imaging was conducted with a source-to-image distance of 100 cm.

The dorsal and lateral decubitus positions resulted in relatively higher radiation doses. Conversely, the AP supine, PA prone, lateral, and AP erect positions exhibited lower ESD and absorbed doses. Statistical analysis revealed no significant differences in ESD and absorbed doses for male gonads across the projections. Additionally, an increase in kVp correlated with a reduction in both ESD and absorbed dose.

This study emphasizes the critical importance of optimizing kVp settings to minimize radiation exposure to male gonads during abdominal X-ray examinations. It also emphasizes the significant influence of radiographic projections on radiation dose, advocating for careful selection of projections to enhance patient safety. These findings contribute to advancing radiological practices, reducing unnecessary radiation exposure, and improving patient care standards.

## Introduction

Medical imaging is a critical field of study that aids in diagnosing various pathologies. Advances in modern techniques have significantly improved image quality, thereby enhancing diagnostic accuracy and broadening the scope of applications in healthcare [1]. However, the International Atomic Energy Agency [2] emphasizes that several factors must be justified before examining, such as the radiation dose and physical parameters involved.

For abdominal X-rays, the procedure generates images of the abdominal cavity using a low dose of ionizing radiation [3]. The most common positions for abdominal radiographs are the anteroposterior (AP) supine, posteroanterior (PA) prone, and lateral projections. A standard abdominal radiograph typically encompasses the area between the diaphragm and the pubic symphysis [4]. Additionally, abdominal radiographs are among the most frequently requested imaging procedures in radiology departments. Variations in projections are often necessary to address specific pathological conditions. For instance, an AP supine projection captures X-rays passing from front to back while the patient lies on their back, providing a straightforward, broad view of the abdomen. Erect projection is useful for visualizing air-fluid levels, which are not visible in a supine position. The decubitus position involves the patients lying on their side, which can aid in detecting specific pathologies [5]. These projections offer distinct benefits, such as providing adequate diagnostic information while being non-invasive, cost-effective, and quick. However, excessive radiation exposure remains a concern, as even low doses carry a small cancer risk. For instance, acute doses above 50 mSv are strongly associated with cancer risk, and doses above 5 mSv show evidence of increased risks [6].

James and Kelly (2013) [7] reported that abdominal radiographs were historically performed in both supine and erect positions. However, this practice has been largely phased out due to concerns over the high radiation dose often associated with these techniques. Further, the Royal College of Radiologists now recommends a single supine abdominal radiograph for patients presenting with acute abdomen. In cases of clinical suspicion of intra-abdominal perforation, an erect chest radiograph may also be performed.

Bowen et al. (2007) [8] estimated the entrance surface dose (ESD) for the male gonad during an AP abdominal X-ray to be approximately 0.17 mGy. Prominent organizations such as the National Council on Radiation Protection and Measurements [9], the International Commission on Radiological Protection [10], and the United Nations Scientific Committee on the Effects of Atomic Radiation [11] have extensively studied ESD for various examinations and provided guidance on radiation exposure limits. These organizations have highlighted the significant role of diagnostic X-ray radiology in human radiation exposure, which accounts for 99% of all man-made radiation exposure [12].

Radiation dose-related concerns have long been a major issue, particularly among the public. It is, therefore, crucial for professionals involved in radiation-related practices to develop a shared understanding of dose quantities, their magnitudes, and the associated risks. Reducing radiation doses to sensitive areas, such as the gonads, is especially important when imaging the abdominal region. For instance, using the PA position instead of the AP position in abdominal radiography can result in a 76% reduction in testicular doses for male patients [13].

Scattered photons, a byproduct of radiation interaction with a medium, pose significant challenges in radiography, particularly problematic in abdominal radiography. It occurs when the X-ray beam is deflected, producing less penetrating rays that degrade image quality, obscure realistic anatomical renderings, and increase radiation exposure to both patients and healthcare workers [14-16]. The interactions responsible for generating scattered radiation in radiography primarily occur within the patient [17]. Strategies such as shielding sensitive areas can effectively reduce scattered radiation. Rosyida and Ekaratri (2018) [18] emphasized the importance of employing protective shielding to mitigate scattered radiation during examinations. Furthermore, proper collimation and patient positioning are essential to minimize scatter radiation and comply with the "as low as reasonably achievable" (ALARA) principle.

Gonadal shielding, introduced over 60 years ago, has been a standard practice to protect the reproductive organs, especially in pediatric imaging using ionizing radiation [19]. However, recent studies question the necessity of gonadal shielding in medical X-ray imaging. Samara et al. (2022) [20] argued that patient shielding may no longer be essential as its effectiveness is increasingly debated. Moreover, they highlight that shielding may negatively affect image quality, rendering its continued use potentially counterproductive.

The absorbed dose quantifies energy deposited in tissue by ionizing radiation, influencing both deterministic (short-term) and stochastic (long-term) effects [21]. Scattered radiation, caused by photon interaction with matter, degrades image quality, increases exposure, and poses risks to patients and workers [18]. Strategies to reduce scatter include precise collimation and shielding, particularly in abdominal imaging. Research shows that proper measurement of absorbed and ESDs helps estimate stochastic risks, guiding dose optimization and minimizing patient exposure [22].

Radiation dose to radiosensitive organs, particularly the gonads, is often overlooked during abdominal examinations. This oversight is significant given the routine requirement of abdominal radiographs in cases of abdominal injuries. The gonad dose, in particular, should not be disregarded due to its potential long-term effects. Limited research has compared gonad doses across various projections, such as AP supine, PA prone, lateral abdominal, erect abdomen, lateral decubitus, and dorsal decubitus. Most existing studies focus on comparing basic and alternative procedures, such as AP supine and PA prone views.

The present study aimed to address this gap by measuring male gonad doses across various abdominal projections at different kilovoltage peak (kVp) settings and a constant milliampere-seconds (mAs) value using an optically stimulated luminescence dosimeter (OSLD). The process involves placing the nanoDot OSLD (Landauer, Inc., Glenwood, IL, USA) on the surface of the phantom corresponding to the location of gonads for ESD measurements and inside the phantom relevant to the location of gonads for absorbed dose measurements.

## Materials and methods

**Figure 1:**
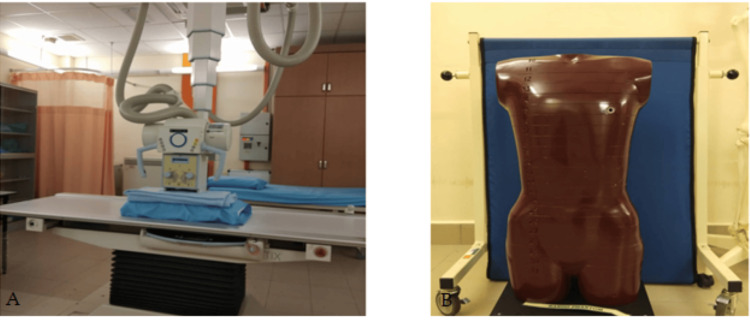
(A) Imaging system and (B) RANDO phantom employed in this study

The X-ray system used in this research is the Siemens Multix Top (Siemens Healthcare Inc., Erlangen, Germany). The Alderson RANDO phantom (The Phantom Laboratory, Salem, New York, USA) is widely regarded as a reliable tool for simulating the human body, particularly in X-ray studies (Figure 1). In this investigation, a male anthropomorphic RANDO phantom was used, representing human X-ray absorption and scattering. The study focused on the abdomen region, where X-ray exposure occurred. The phantom consists of 36 sections (numbered 0 to 35), with sections 0 to 34 having a thickness of 2.5 cm and section 35 (the pelvic floor) being approximately 9 cm thick. All sections, except 0 and 35, include holes for dosimeter placement.

The RANDO phantom was positioned on the table bucky for various abdominal projections: AP supine, PA prone, lateral abdominal, lateral decubitus, and dorsal decubitus. For the erect bucky, the phantom was positioned in the AP standing view. A 35 cm x 43 cm imaging plate was used for each projection. The kVp was varied between 81, 83, and 85, while the mAs was fixed at 32 for all projections. The central ray (CR) direction varied depending on the projection. The source-to-image distance was fixed at 100 cm for all projections. A stationary grid with a grid ratio of 8:1 was used, and the automatic exposure control was turned off for the lateral decubitus and dorsal decubitus projections.

The male gonad’s ESD and the absorbed dose by the RANDO phantom were measured using a nanoDot OSLD. The dosimeter was placed outside the phantom at the gonadal area to measure the ESD and inside the phantom, between partitions 34 and 35, to measure the absorbed dose. These measurements were repeated for each of the different abdominal positions: AP supine, PA prone, lateral abdominal, lateral decubitus, and dorsal decubitus. Furthermore, pre-irradiation readings of nanoDot OSLDs were recorded to be subtracted from post-irradiation readings, ensuring accurate dose measurements.

**Figure 2:**
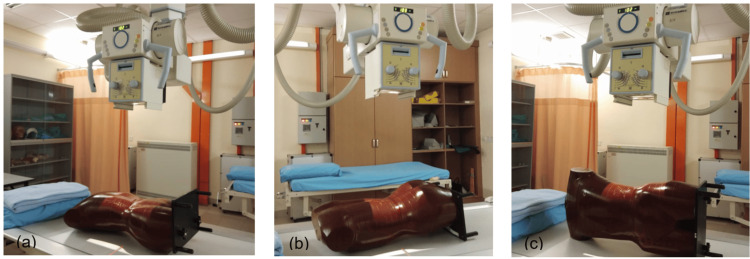
(a) AP supine abdomen, (b) PA prone abdomen, and (c) lateral abdomen projections AP: anteroposterior, PA: posteroanterior

**Figure 3:**
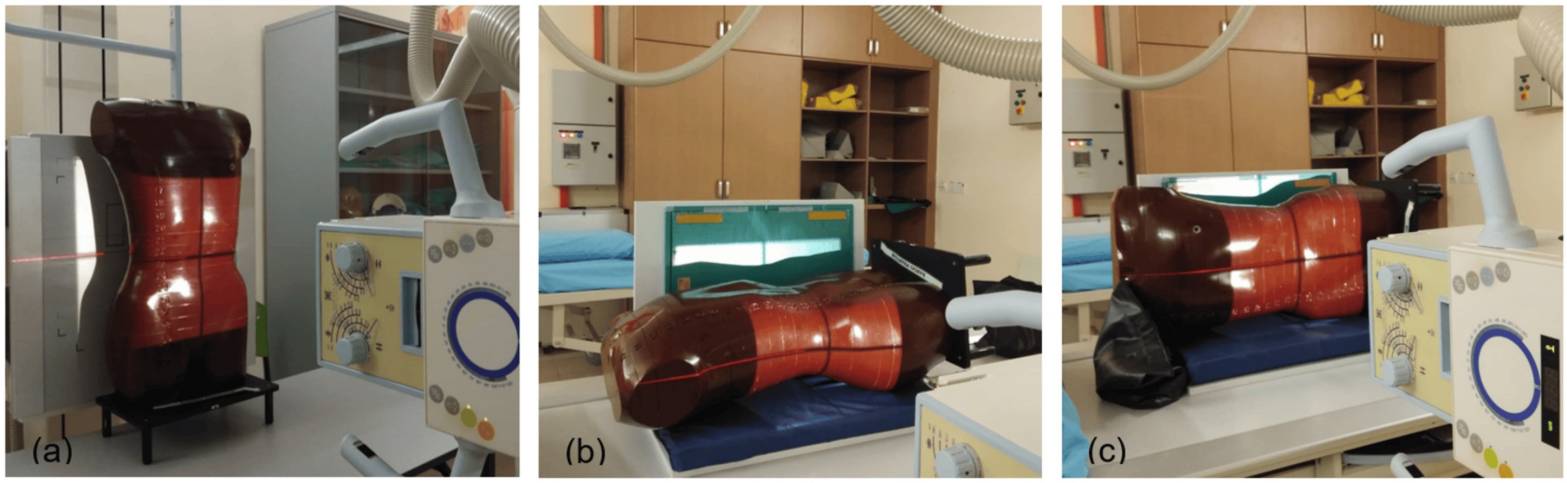
Three different projections: (a) AP erect abdomen, (b) dorsal decubitus abdomen, and (c) lateral decubitus abdomen AP: anteroposterior

For the AP supine and PA prone projections, the CR was perpendicular to the image receptor, centered above the level of the iliac crest. For the lateral abdominal projection, the CR was positioned at the level of the iliac crest to the mid-coronal plane. A horizontal beam was used in the lateral decubitus and dorsal decubitus positions, centered on the image receptor 2 inches above the iliac crest. Figures 2-3 illustrate the positioning setups for these procedures.

Statistical analysis

SPSS Statistics version 28 (IBM Corp. Released 2021. IBM SPSS Statistics for Windows, Version 28.0. Armonk, NY: IBM Corp.) was employed to analyze the data. The Kruskal-Wallis test was used to examine significant differences between the mean ESD and the absorbed dose for various projections during abdominal X-ray examinations at different kVp settings. A p-value of less than 0.05 was considered statistically significant.

## Results

Assessment of male gonadal entrance skin dose across various radiographic projections

**Figure 4:**
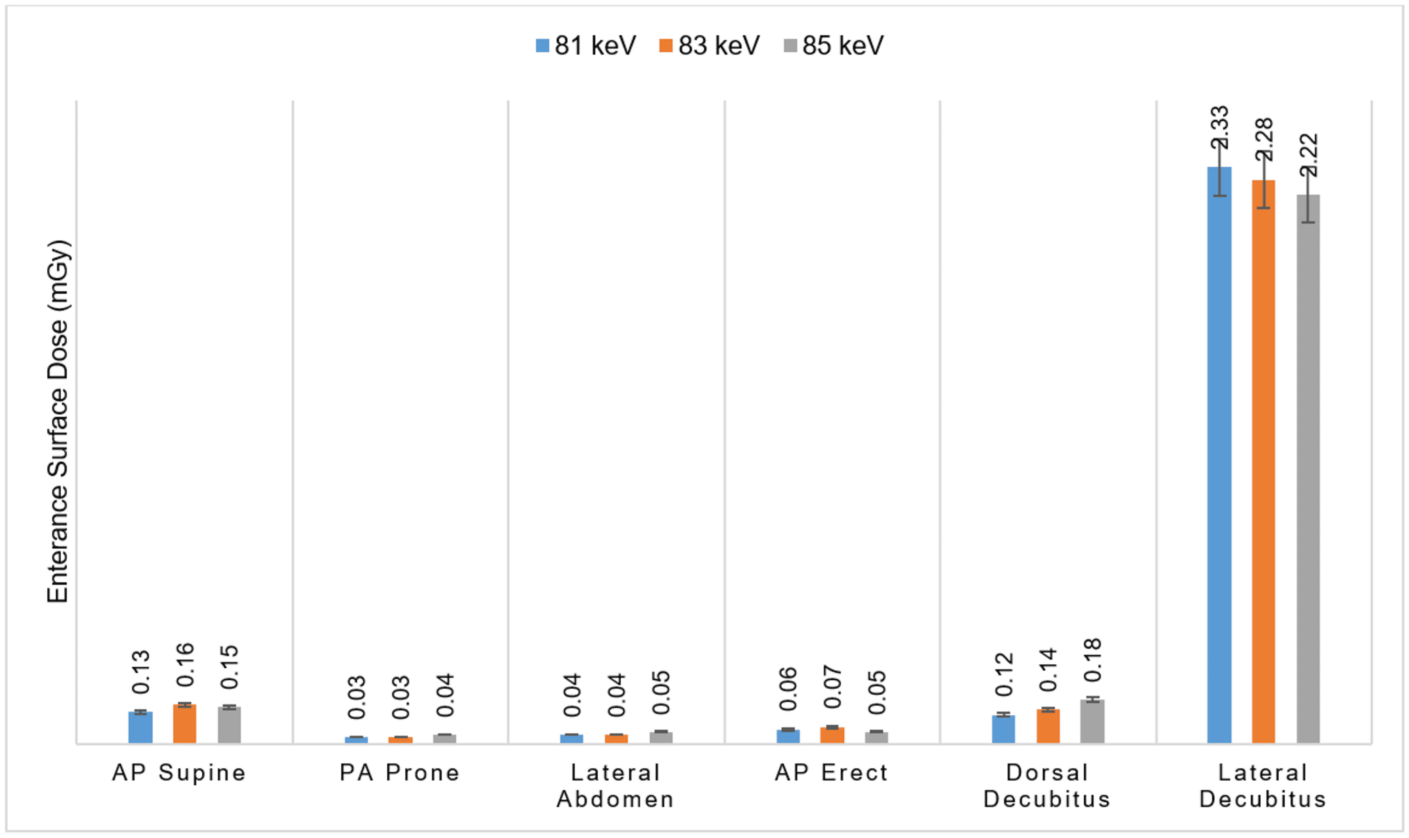
Measured ESD to male gonads at different kVp and projections while constant mAs Data are presented as mean±SD, with significance defined as a p-value of <0.05. ESD: entrance surface dose, kVp: kilovoltage peak, mAs: milliampere-seconds, AP: anteroposterior, PA: posteroanterior

The study assessed the radiation doses to male gonads (ESD) across various abdominal X-ray projections. RANDO phantom was exposed at 81, 83, and 85 kVp settings, with a constant exposure value of 32 mAs. ESD was evaluated for six projections: AP supine abdomen, PA prone abdomen, lateral abdomen, AP erect abdomen, dorsal decubitus abdomen, and lateral decubitus abdomen. Figure 4 presents the male gonadal ESD results, which were statistically analyzed using the Kruskal-Wallis test in SPSS Statistics.

Among the projections, the lateral decubitus abdomen had the highest ESD values for gonad dose: 2.33, 2.28, and 2.22 mGy for 81, 83, and 85 kVp, respectively. These values showed a slight decrease with increasing kVp. In contrast, other projections, such as the AP supine abdomen, PA prone abdomen, lateral abdomen, AP erect abdomen, and dorsal decubitus abdomen, exhibited significantly lower average ESD gonad dose values. In the PA prone abdomen, consistently lowest gonadal radiation doses were measured: 0.03, 0.02, and 0.04 mGy for 81, 83, and 85 kVp, respectively.

Assessment of male gonadal absorbed dose across different radiographic projections

**Figure 5:**
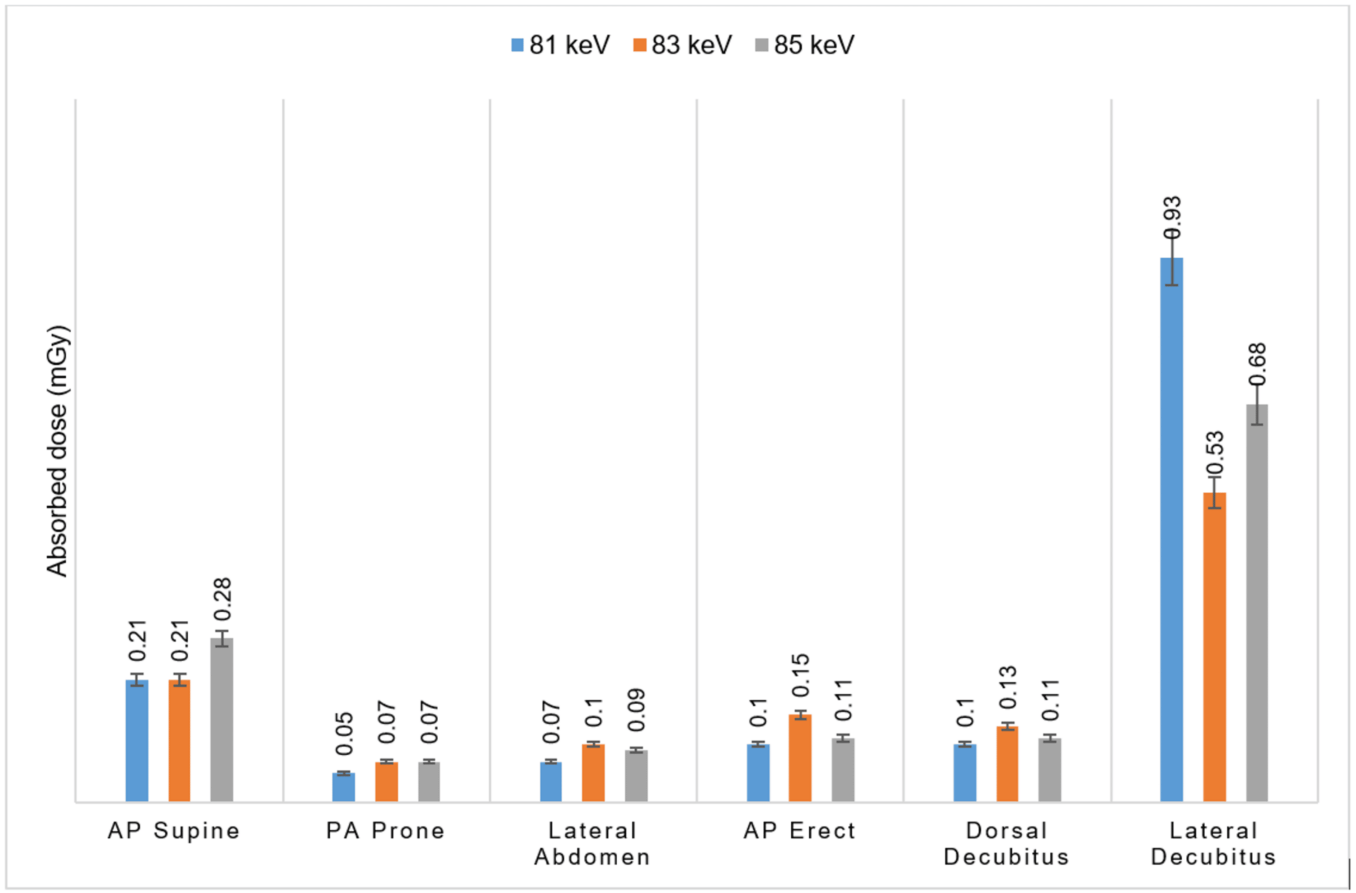
Measured absorbed dose to male gonads at different kVp and projections while constant mAs Data are presented as mean±SD, with significance defined as a p-value of <0.05. kVp: kilovoltage peak, mAs: milliampere-seconds, AP: anteroposterior, PA: posteroanterior

Absorbed dose data, illustrated in Figure 5, were measured for the same six projections at 81, 83, and 85 kVp. The lateral decubitus abdomen projection showed the highest absorbed doses to gonads, with values of 0.93, 0.93, and 0.68 mGy at 81, 83, and 85 kVp, respectively.

The AP supine abdomen, often used as a routine projection, recorded the second-highest absorbed dose to gonads: 0.21, 0.21, and 0.28 mGy for the same kVp values. Other positions, including the PA prone abdomen, lateral abdomen, AP erect abdomen, and dorsal decubitus abdomen, showed comparable and relatively low absorbed doses.

Comparison of male gonadal dose: entrance surface dose versus absorbed dose

Figures 4-5 illustrate the average ESD and absorbed dose to male gonads across the different projections and kVp settings at fixed mAs. For the AP supine abdomen, PA prone abdomen, lateral abdomen, and AP erect abdomen, the absorbed dose exceeded the ESD, particularly at 85 kVp. In contrast, the ESD was higher than the absorbed dose for the dorsal decubitus abdomen and lateral decubitus abdominal projections.

The dorsal decubitus abdomen recorded its highest ESD at 83 kVp, while the lateral decubitus abdomen consistently declined ESD from 81 to 85 kVp. Overall, the data suggest that increasing kVp values generally lead to reduced ESD and absorbed doses.

Analysis of entrance surface dose and absorbed dose

The Kruskal-Wallis test assessed the statistical significance of variations in ESD and absorbed doses across different projections and kVp settings. For ESD, no statistically significant differences were observed (p=0.949). Similarly, for the absorbed dose, the differences were also not statistically significant (p=0.895). Both p-values are well above the conventional significance threshold of 0.05.

## Discussion

According to Shahbazi-Gahrouei (2006) [23], assessing the radiation dose received by patients undergoing X-ray examinations is essential to evaluating the associated risks. The patient's radiography dose depends on the ESD and the sensitivity of the irradiated organs and tissues during the procedure. Numerous studies have been conducted worldwide to evaluate ESD, with results compared against exposure levels recommended by regulatory organizations such as the International Commission on Radiation Protection [10], the National Radiological Protection Board [24], and the International Atomic Energy Agency [2].

As part of the legislative drafting process, measuring ESD and providing guidance to the healthcare community has been recognized as a practical approach to reducing patient radiation exposure in diagnostic radiography. The ESD refers to the absorbed dose to the patient’s skin at the center of the irradiated area. Measurements of ESD values for routine radiographic examinations have been conducted, providing a useful parameter for estimating the radiation dose received by patients. This, in turn, serves as an approximate indicator of stochastic radiation risks [22].

Furthermore, the absorbed dose measures energy deposition in tissues by ionizing radiation, which is relevant to understanding both stochastic (e.g., cancer risks) and deterministic effects [21,25]. Absorbed dose, a fundamental measure of radiation, quantifies energy deposition by ionizing radiation in a medium. It is relevant to all radiation types, absorbing media, and biological targets. The absorbed dose correlates with ionization events, indicating physical tissue damage [25]. Expressed in gray (Gy), the absorbed dose is critical for assessing radiation exposure risks in medical, workplace, and environmental settings [26]. The ESD and absorbed dose are both critical parameters in radiation dose assessment.

The findings of the current study reveal that different radiographic projections can impact the ESD in the gonad area. The results show that positioning the phantom in the lateral decubitus leads to higher ESD values than the other projections. This projection utilizes a horizontal beam with a stationary grid, differing significantly from other vertical beam projections. A horizontal beam has several advantages in diagnosing specific pathologies. Hooker et al. (2008) [27] noted that the addition of decubitus images increases the number of diagnostically useful investigations, including the ability to identify or rule out intussusception. According to the authors, a left-side-down decubitus view should be included in the initial evaluation of patients suspected of having intussusception, especially when the supine view is inconclusive. Adding the left lateral decubitus image to an abdominal radiograph improves diagnostic sensitivity and specificity for intussusception, increasing the percentage of diagnostically determinative studies from 36.2% to 67.4% [27]. Furthermore, the data analysis shows that ESD values decreased as the kVp exposure increased. This is because higher kVp results in greater penetration of the phantom, which also leads to increased scattered radiation.

**Figure 6:**
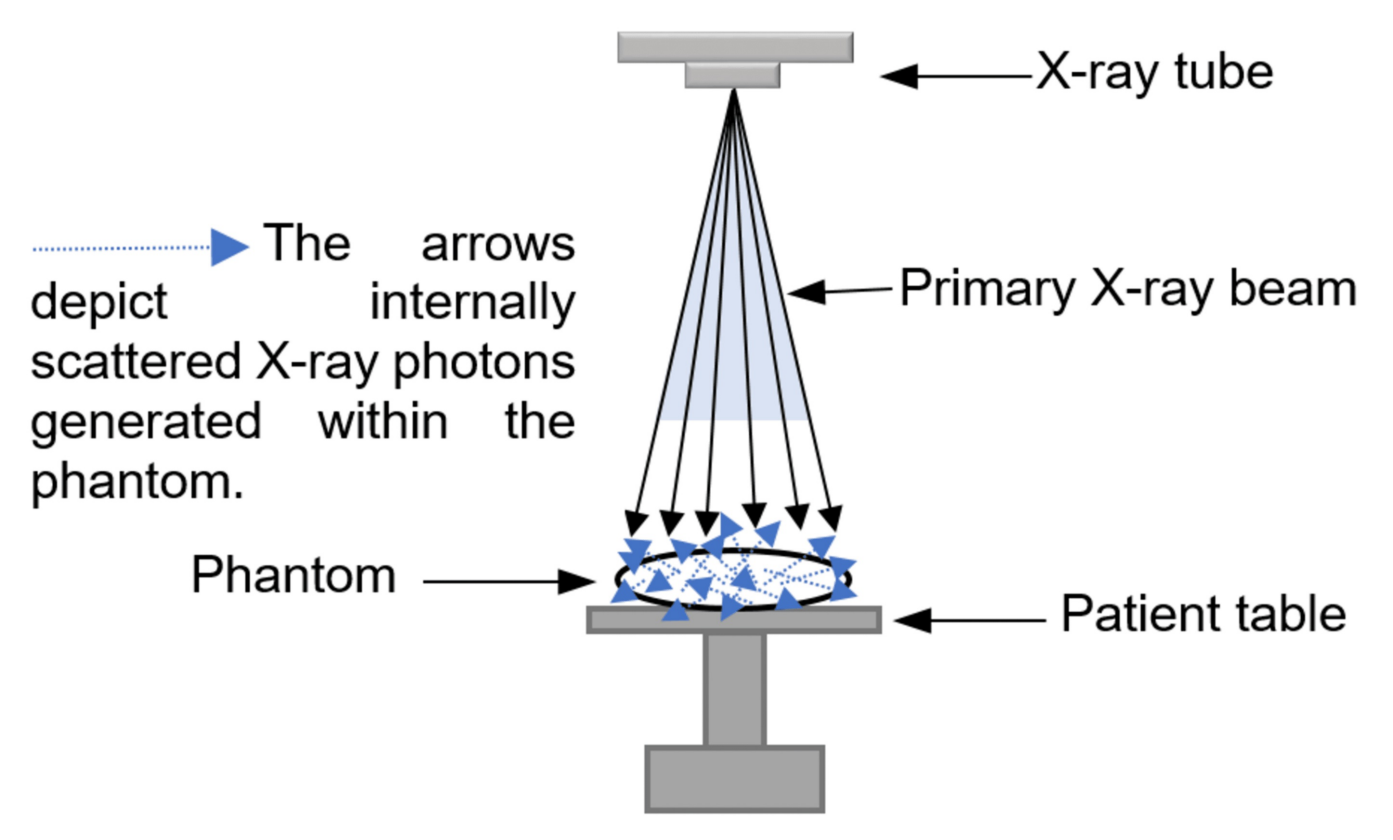
Generation of internal scatter within the phantom Image Credit: Author

In the present study, the absorbed dose was measured by placing the nanoDot OSLD in the phantom section corresponding to the male gonads. Data analysis revealed that the absorbed dose was higher for projections such as the AP supine abdomen and lateral decubitus. In contrast, PA prone abdomen, lateral abdomen, AP erect abdomen, and dorsal decubitus positions showed reduced absorbed dose. These radiographic projections involved varied beam directions, such as vertical and horizontal beams. In this study, for projections such as the AP supine abdomen, PA prone abdomen, and lateral abdomen, the phantom was placed on top of the table bucky, with the CR aligned to a vertical beam. These projections are typically used for routine patient imaging. When the radiation exits the patient's body, it hits the table bucky. The grid absorbs some scattered radiation, while some is reflected onto the patient’s body. The interactions responsible for generating scattered radiation in radiography primarily occur within the patient [17], as illustrated in Figure 6. Compared to the primary beam, backscatter energy is lower, causing the body to absorb more of the energy, thus increasing the absorbed dose. The study revealed that increasing the kVp led to a decreased absorbed dose. If the dose is delivered in a short time in a single high-dose exposure, there is insufficient time for radiation damage to be repaired, and the damage per unit dose is high. Conversely, when the total dose is delivered at a reduced rate, there is more time for repair, and the damage per unit dose is lower [28].

Sinno-Tellier et al. (2006) [29] reported a gonadal radiation dose of 0.70 mGy, while Bowen et al. (2007) [8] measured a dose of 0.17 mGy for AP abdomen, supine abdomen, and erect abdomen projections. Hajizadeh et al. (2007) [30] observed an average gonadal dose of 0.442 mGy across 10 counties in the Khorasan province of Iran. Similarly, Chaparian et al. (2014) [13] reported gonadal doses of 0.152 mGy for AP projections and 0.037 mGy for PA projections. The NCRP [9], in Statement No. 13, cited a gonadal dose of 1.81 mGy during abdominopelvic radiography.

In the current study, the ESD ranged from 0.03 to 2.33 mGy, while the absorbed dose varied between 0.05 and 0.93 mGy across various radiographic projections. These values are generally lower than the doses reported in the studies above, except for the ESD in the lateral decubitus projection, which was comparatively higher.

Minimizing radiation exposure is essential, and this study reinforces the importance of adhering to the ALARA principle in abdominal radiography. Advancements in diagnostic imaging technology, including the integration of artificial intelligence, computed radiography, faster image receptors, enhanced X-ray beam filtration, and improved X-ray generators, have significantly reduced gonadal doses during abdominal radiography compared to doses recorded a couple of decades ago.

Despite these advancements, further research is needed to reduce gonadal exposure, particularly by addressing internal scatter radiation. Before interacting with the patient, the X-ray beam consists predominantly of primary X-rays, with minimal interaction with air. However, as the beam passes through the patient’s body, attenuation reduces the number of primary X-rays and generates scattered X-rays. This results in an increasing scatter-to-primary ratio as the beam penetrates deeper.

The scatter-to-primary ratio is lowest near the body's surface where the X-ray beam enters (e.g., around the testes), higher at intermediate depths such as the ovaries, and reaches its peak where the beam exits the body. Additionally, the scatter-to-primary ratio increases with patient size, as noted by the NCRP [9]. Addressing these factors through continued innovation and optimized protocols can further enhance radiation safety in abdominal radiography.

Limitations of the study

The placement of nanoDot dosimeters in the phantom was suboptimal due to the absence of a designated region for the male gonads. Consequently, the dosimeters were positioned on a nearby partition, which may have measured radiation exposure to surrounding soft tissues rather than directly to the gonads. Furthermore, inconsistencies in the placement of the nanoDot OSLDs could have introduced measurement errors.

## Conclusions

This study highlights the importance of effective radiation management in abdominal imaging to enhance patient safety. The findings showed no significant difference between ESD and absorbed dose, though variations were observed based on radiographic projections. The ESD and absorbed dose were significantly higher for the lateral decubitus projection than other projections.

It is impossible to eliminate scattered radiation during radiographic procedures completely. Managing internally scattered photons presents a greater challenge than addressing scatter outside the patient’s body. Consequently, further research is crucial to reduce gonadal exposure, particularly by mitigating the effects of internal scatter radiation. Minimizing unnecessary radiation exposure to the male gonads during abdominal radiography requires careful consideration of exposure factors, radiographic projections, patient positioning, and precise collimation. Strict adherence to the ALARA principle is essential to ensuring optimal radiation protection.
